# Low-dose abdominopelvic computed tomography in patients with lymphoma: An image quality and radiation dose reduction study

**DOI:** 10.1371/journal.pone.0272356

**Published:** 2022-08-11

**Authors:** Sungjin Yoon, Kwai Han Yoo, So Hyun Park, Hawk Kim, Jae Hoon Lee, Jinny Park, Seong Ho Park, Hwa Jung Kim

**Affiliations:** 1 Department of Radiology, Gil Medical Center, Gachon University College of Medicine, Incheon, Republic of Korea; 2 Department of Internal Medicine, Gachon University Gil Medical Center, Gachon University College of Medicine, Incheon, Korea; 3 Department of Radiology and Research Institute of Radiology, University of Ulsan College of Medicine, Asan Medical Center, Seoul, Korea; 4 Department of Clinical Epidemiology and Biostatistics, Asan Medical Center, Ulsan University College of Medicine, Seoul, Korea; Northwestern University Feinberg School of Medicine, UNITED STATES

## Abstract

This study aimed to evaluate image quality, the detection rate of enlarged lymph nodes, and radiation dose exposure of ultralow-dose and low-dose abdominopelvic computed tomography (CT) in patients with lymphoma. Patients with lymphoma who underwent abdominopelvic CT using dual-source scanner were retrospectively recruited from a single center. CT images were obtained at 90 kVp dual-source mode reformatted in three data sets using the advanced modelled iterative reconstruction algorithm: 100% (standard-dose CT), 66.7% (low-dose CT), and 33.3% (ultralow-dose CT). Two radiologists analyzed subjective image quality and detection of abdominal enlarged lymph nodes on ultralow-dose, low-dose, and standard-dose CT blindly and independently. The results were compared with reference standards. Three readers (two radiologists and one hematologist) reviewed overall image quality and spleen size. In total, 128 consecutive CT scans (77 complete response, 44 partial response, 6 progressive disease, and 1 initial evaluation) from 86 patients (64 B-cell lymphoma, 14 T/NK-cell lymphoma, and 8 Hodgkin’s lymphoma cases) were assessed. The enlarged lymph node-based detection rates for two readers were 97.0% (96/99) and 94.0% (93/99) on standard-dose CT, 97.0% (96/99) and 94.0% (93/99) on low-dose CT, and 94.0% (93/99) and 89.9% (89/99) on ultralow-dose CT. Overall image quality was 3.8 ± 0.5, 3.9 ± 0.5, and 4.1 ± 0.5 on ultralow-dose CT; 4.7 ± 0.4, 4.6 ± 0.5, and 4.8 ± 0.3 on low-dose CT; and 4.8 ± 0.4, 4.7 ± 0.4, and 4.9 ± 0.2 on standard-dose CT, according to two radiologists and one hematologist, respectively. Intraclass correlation coefficients of spleen size were 0.90 (95% confidence interval [CI], 0.87–0.93), 0.91 (95% CI, 0.88–0.93), and 0.91 (95% CI, 0.88–0.93) on ultralow-dose, low-dose, and standard-dose CT, respectively. Mean effective radiation doses of standard-dose, low-dose, and ultralow-dose CT were 5.7 ±1.8 mSv, 3.8 ± 1.2 mSv, and 1.9 ± 0.6 mSv, respectively. Our findings suggest that ultralow-dose and low-dose CT, even with radiation doses reduced by 66.7% and 33.3%, respectively, maintained adequate image quality. These imaging modalities may be employed for follow-up lymphoma evaluation in consideration of the long surveillance periods.

## Introduction

Lymphomas account for 3.4% of all malignancies worldwide and consist of heterogeneous subtypes (i.e., non-Hodgkin’s lymphomas and Hodgkin’s lymphoma) [1, 2]. The majority of lymphomas involve the lymph nodes and extralymphatic organs, and occur in young adults and pediatric patients. A proportion of lymphomas are considered potentially curable diseases due to improvements in treatment protocols [3]. The Lugano classification is used for lymphoma staging and response assessment, and imaging modalities such as positron emission tomography (PET)/CT and computed tomography (CT) play key roles in response assessment and surveillance to evaluate lymph nodes and spleen [4–7]. Due to their long life expectancy, patients with lymphoma require multiple CT examinations to evaluate treatment response and surveillance after treatment. The cumulative radiation exposure of repetitive CT examinations in pediatric and young adult patients may increase baseline cancer risk [8, 9]. Therefore, dose reduction techniques for CT can be useful for patients with lymphoma in consideration of the long surveillance periods.

Several studies have attempted low-dose CT to evaluate Hodgkin’s lymphoma in the thorax [10] and in patients undergoing staging or restaging of lymphoma [11, 12]. Generally, a reduction in radiation dose is related to increased image noise and decreased image quality, which may negatively impact diagnostic performance. However, in patients with lymphoma, particularly during follow-up after treatment, measurement of pre-existing lesions or improvements in lymphoma involvement constitute a major component of CT assessments. In this regard, low-dose CT with reduced image quality may be sufficient for follow-up evaluation.

Recent advances in CT techniques have contributed to a reduction in radiation dose, such as the implementation of automatic adjustment of tube potential, automated tube current modulation, and iterative reconstruction (IR) [13, 14]. A new third generation of IR was recently developed based on statistical-based to model-based IR [14–16]. Advanced modelled IR (ADMIRE; Siemens Healthcare, Forchheim, Germany) [17] is a model-based IR that permits a large reduction in image noise in raw data and additional dose reduction with improved spatial resolution.

Dual-energy CT with dual-source scanners can be used to compare standard and low-dose CT images by separating and combining data from each tube without the need for additional examinations [18, 19]. In this study, we evaluated radiation dose exposure and image quality of low-dose and standard-dose CT to compare the detection rates of enlarged lymph nodes of low-dose and standard-dose CT for abdominal lymphoma evaluation and post-treatment follow-up in patients with lymphoma.

## Materials and methods

### Ethics approval

Approval for this retrospective study was obtained from the institutional review board (GAIRB2021-237) of the Gil Medical Center. All CT images were acquired using standard-dose CT scan, without additional radiation exposure. Thus, the informed consent was waived because of the retrospective nature of the study.

### Study participants

**Fig 1:**
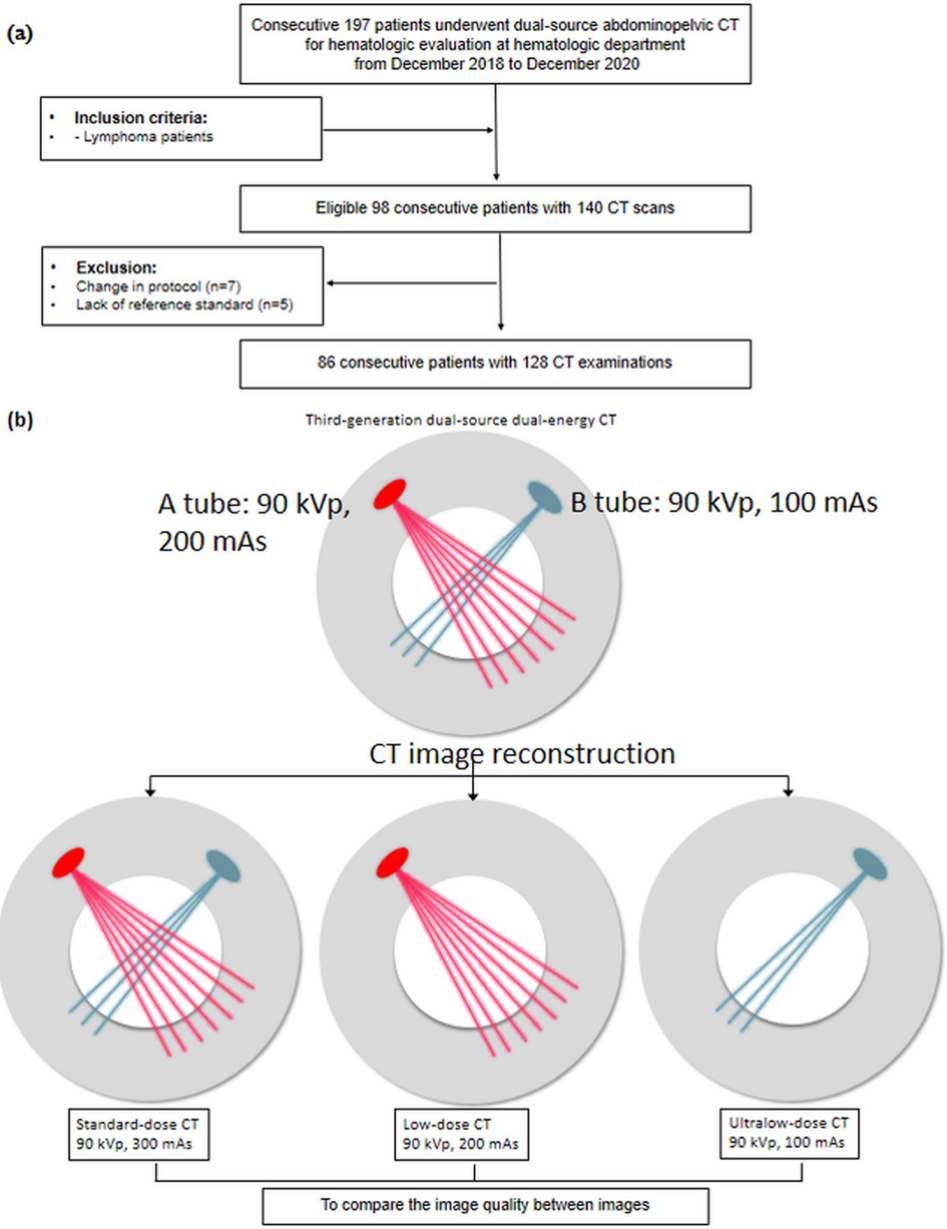
Flow diagram of patients. (a) Inclusion flow chart and (b) computed tomography (CT) examinations and reconstruction methods.

In total, 197 consecutive patients who underwent dual-source CT scans for hematologic assessment at the hematologic department of Gil Medical Center from December 2018 to December 2020 were enrolled. All patients diagnosed with lymphoma were included. Patients underwent CT for lymphoma evaluation, chemotherapy response, or surveillance after treatment. Among 98 consecutive patients with 140 CT scans who were eligible, 12 patients were excluded due to a protocol change (n = 7) and lack of a reference standard (n = 5). A final total of 86 consecutive patients with 128 CT examinations were included in this study (Fig 1A). The study period, from December 2018 to March 2019, partially overlapped with that of a previous dual-energy study conducted in the same institution [20]. However, the previous study only included patients from the oncologic department. Therefore, there were no overlapping patients between the two studies.

### CT technique

Contrast-enhanced abdominopelvic CT examination was acquired above the dome of the diaphragm and below the symphysis pubis. All patients received intravenous injection of 1.5 mL/kg of iopamidol (Pamiray 300; Dongkook Pharm., Korea), up to a maximum dose of 120 mL. The injection was delivered using a power injector at an injection rate of 4 mL/s and fixed injection duration of 75 s. CT scans were achieved at a fixed tube potential of 90 kVp using a third-generation dual-source CT scanner (SOMATOM Force, Siemens Healthcare, Forchheim, Germany) in dual-source mode with tube detector A (reference tube current: 100 mAs) and B (reference tube current: 200 mAs), using tube current dose modulation (CARE dose 4D; Siemens Healthcare) and the ADMIRE algorithm (Fig 1B and Table 1). We used the ADMIRE algorithm at a strength level of 2 out of 5, with an axial slice thickness of 5 mm and coronal slice thickness of 3 mm.

**Table 1:** Reconstruction parameters.

	Standard-dose	Low-dose	Ultralow-dose
Radiation dose exposure	100%	66.7%	33.3%
Tube detector	Mix of detector A and B	Tube detector A	Tube detector B
Kilovolt (kV)	90	90	90
Automated tube voltage selection	Off	Off	Off
Reference tube current (mAs)	300 (100%)	200 (66.7%)	100 (33.3%)
Automated tube current modulation	On	On	On
Thickness of axial image	5 mm	5 mm	5 mm
Thickness of coronal image	3 mm	3 mm	3 mm
Pitch	1.15	1.15	1.15
Rotation time (sec)	0.5	0.5	0.5

### Qualitative visual image analysis

Two radiologists (S.J.Y and S.H.P, with 5 and 10 years of abdominal radiologic experience radiologists, respectively) reviewed the image analysis, and one hematologist (K.H.Y) reviewed the overall image quality and spleen size only. The images of 384 CT examinations were reviewed independently by three readers in a blinded manner. These interpretations were analyzed in three reading sessions, some of which included one-third of the three CT image sets. The images were reviewed anonymously, and the order of review was randomized with a 1-month washout period between sessions. Lymphoma involvement was evaluated based on the Lugano classification [4], with modifications: lymph nodes, spleen, liver, and other sites. An enlarged lymph node was regarded as a short-axis diameter > 1 cm, and splenomegaly was defined as the longest length of the spleen > 12 cm. Suspicious masses or nodules in the adrenal glands or liver were analyzed. Enlarged lymph nodes exhibiting a fatty hilum were considered to indicate a reactive change. We measured the average diameter of lymph nodes (short-axis diameter >0.5 cm in each data set) on standard-dose, low-dose, and ultralow-dose CT. The overall image quality score for assessing CT images was subjectively measured using a 5-point (Table 2).

**Table 2:** Qualitative visual image analysis.

Variables	Analysis
**Organ**	**Positive findings**
Liver	Nodules (except definite hemangiomas or cysts)
Spleen	> 12 cm in length, mass, or nodule
Adrenal gland	Nodules
Lymph nodes	Enlarged, short diameter > 1 cm
**Overall image quality**	1, nondiagnostic quality, extremely severe artifacts, insufficient for diganosis
	2, poor image quality, severe artifacts causing uncertainty
	3, moderate image quality, moderate artifacts with mild restricted evaluation
	4, good quality, slight artifacts with sufficient for diagnosis
	5, excellent image quality, no artifacts

### Quantitative image noise analysis

To achieve objective image quality, regions of interest (ROI) were placed in four regions including psoas muscle, subcutaneous fat in the anterior abdominal wall, right hepatic lobe parenchyma, aorta lumen in L1 vertebral body level with same location in three image sets. The standard deviations (SDs) in Hounsfield units (HU) were measured by a 1–3 cm^2^ ROI by a single-blinded reader (S.J.Y.), as image noise. Mean attenuation values (HU) were measured for each ROI.

### Radiation dose

The volume CT dose index (CTDI_vol_) and dose-length product were documented in the dose page of the scanner. In the calculation of effective dose, tissue-weighting factors for the abdomen was used in millisieverts (mSv) (male, *k* = 0.013; female, *k* = 0.017) and pelvis (male, *k* = 0.010; female, *k* = 0.016) using average values (male, k = 0.012; female, k = 0.017) [21, 22].

### Reference standards

Reference standards comprised PET-CT or MRI within 3 months, interval changes of the lesion compared to serial CTs with patient’s symptoms, and clinician’s judgment based on electronic medical records (EMRs). Response assessment was based on EMRs and was categorized into complete, partial, stable, and progressive disease.

### Statistical analysis

Radiation dose and objective image analyses were compared among the three CT scans using analysis of variance followed by post hoc Bonferroni correction. Subjective image evaluations of the three image analyses were compared using Kruskal-Wallis test adjusted with Monte Carlo simulation. In each image set, the detection rate for enlarged lymph nodes were compared with each other using Generalized Estimating Equations (GEE) with adjustment for multiple comparisons using Bonferroni correction. Interobserver agreement of enlarged lymph node detection among two readers was analyzed using kappa, and interobserver agreement of spleen size among three readers was assessed using intraclass correlation coefficients, defined as follows: 0.01–0.20, slight; 0.21–0.40, fair; 0.41–0.60, moderate; 0.61–0.80, substantial; and 0.81–1, excellent. Statistical significance was set at *P* < 0.05. After post hoc analysis, *P*-values < 0.01 were considered statistically significant. Statistical analyses were performed using SAS version 9.4 (SAS Institute Inc., Cary, NC, USA) for all data analyses.

## Results

### Patient characteristics

The clinical characteristics of the patients are summarized in Table 3. Of the 86 patients with 128 CT scans, 57 were men and 29 were women, with a mean age ± standard deviation of 58.4 ± 16.3 years. Of patients, 33 underwent two or more CT examinations during the study period (25 patients, 2 CT examinations; 8 patients, 3 CT examinations). Among patients, B-cell lymphoma was the most common disease (n = 63, 73.3%), followed by T/NK-cell lymphoma (n = 14, 16.3%) and Hodgkin’s lymphoma (n = 8, 9.3%).

**Table 3:** Clinical characteristics of patients.

Parameter	Value
Number of patients	86
Age (years), mean ± SD	58.4 ± 16.3
Men: women	57: 29
Height (cm)	164.1 ± 8.3
Weight (kg)	66.4 ± 13.6
Effective diameter (cm)	26.6 ± 3.2
BMI (kg/m^2^)	24.5 ± 3.7
< 18.5: thin	2 (2.3)
18.5–24.9: normal	47 (54.7)
25–29.9: overweight	29 (33.7)
30–34.9: moderate obesity	8 (9.3)
35–39.9: severe obesity	0
Histology	
Non-Hodgkin’s lymphoma	78 (90.7)
B-cell lymphoma	63 (73.3)
T/NK-cell lymphoma	14 (16.3)
Hodgkin’s lymphoma	8 (9.3)
Patient class	
Inpatient	5 (5.8)
Outpatient	81 (94.2)
CT examinations	128
Disease status	
Initial	1 (0.8)
Progressive disease	6 (4.7)
Partial response	44 (34.4)
Complete response	77 (60.2)
Reference standard	
PET/CT	46 (35.9)
CT	81 (63.3)
PET/CT and liver MRI	1 (0.8)

Note: Data are presented as number (%), unless indicated otherwise.

### Lesion detection analysis

**Fig 2:**
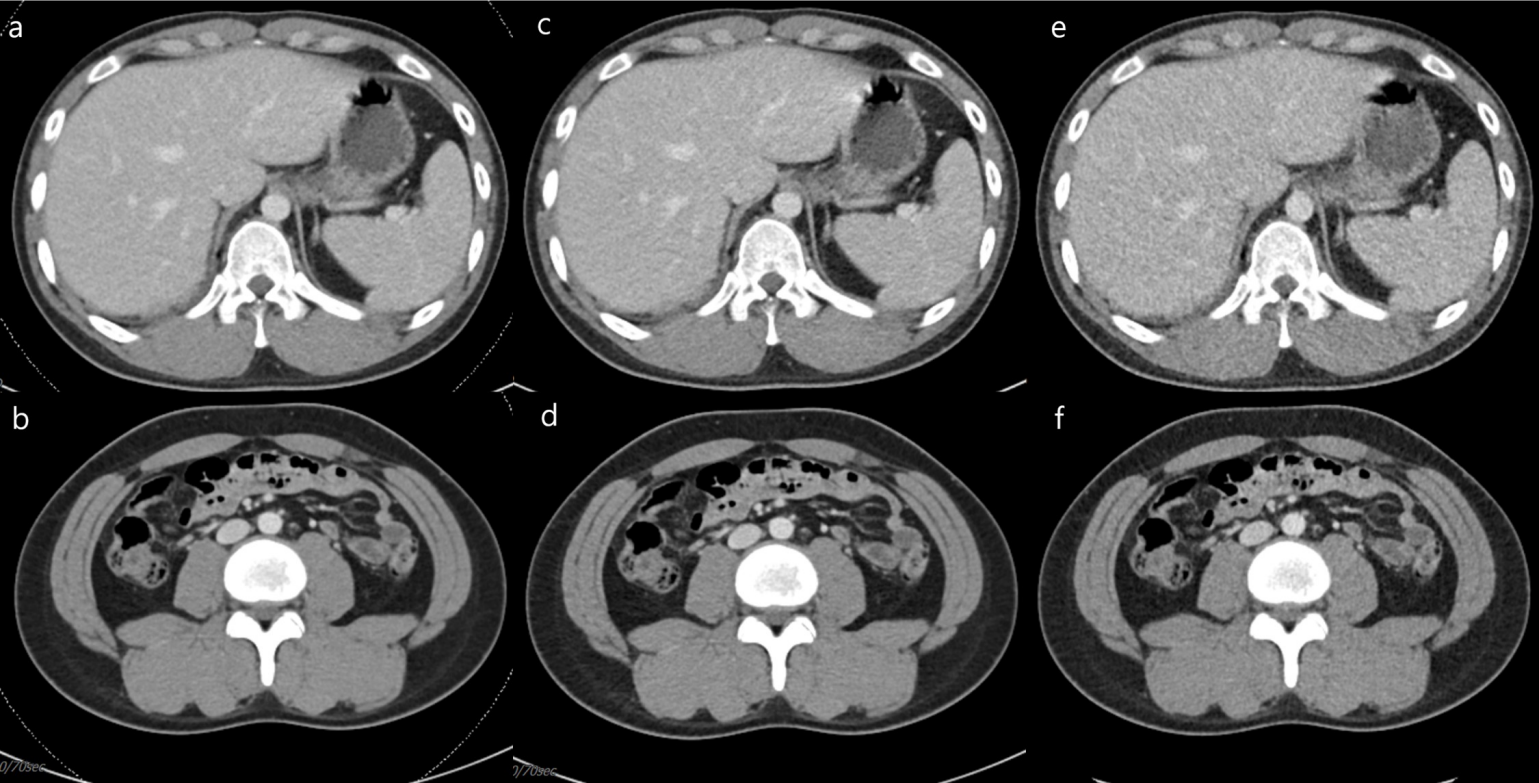
Abdominopelvic computed tomography (CT) images of a 23-year-old woman with Hodgkin’s lymphoma, complete remission state (body mass index, 24.2 kg/m^2^; effective diameter, 24.0 cm). The three different types of CT images, acquired according to the radiation dose (a-b: standard-dose CT, 5.6 mSv; c-d: low-dose CT, 3.7 mSv; e-f: ultralow-dose CT, 1.8 mSv), show the absence of enlarged lymph nodes and splenomegaly. Identical reports of the aforementioned features were also obtained from two radiologists. The overall image quality score was 4 for the ultralow-dose CT images and 5 for the low-dose and standard-dose CT images, according to three readers (two radiologists and one hematologist).

**Fig 3:**
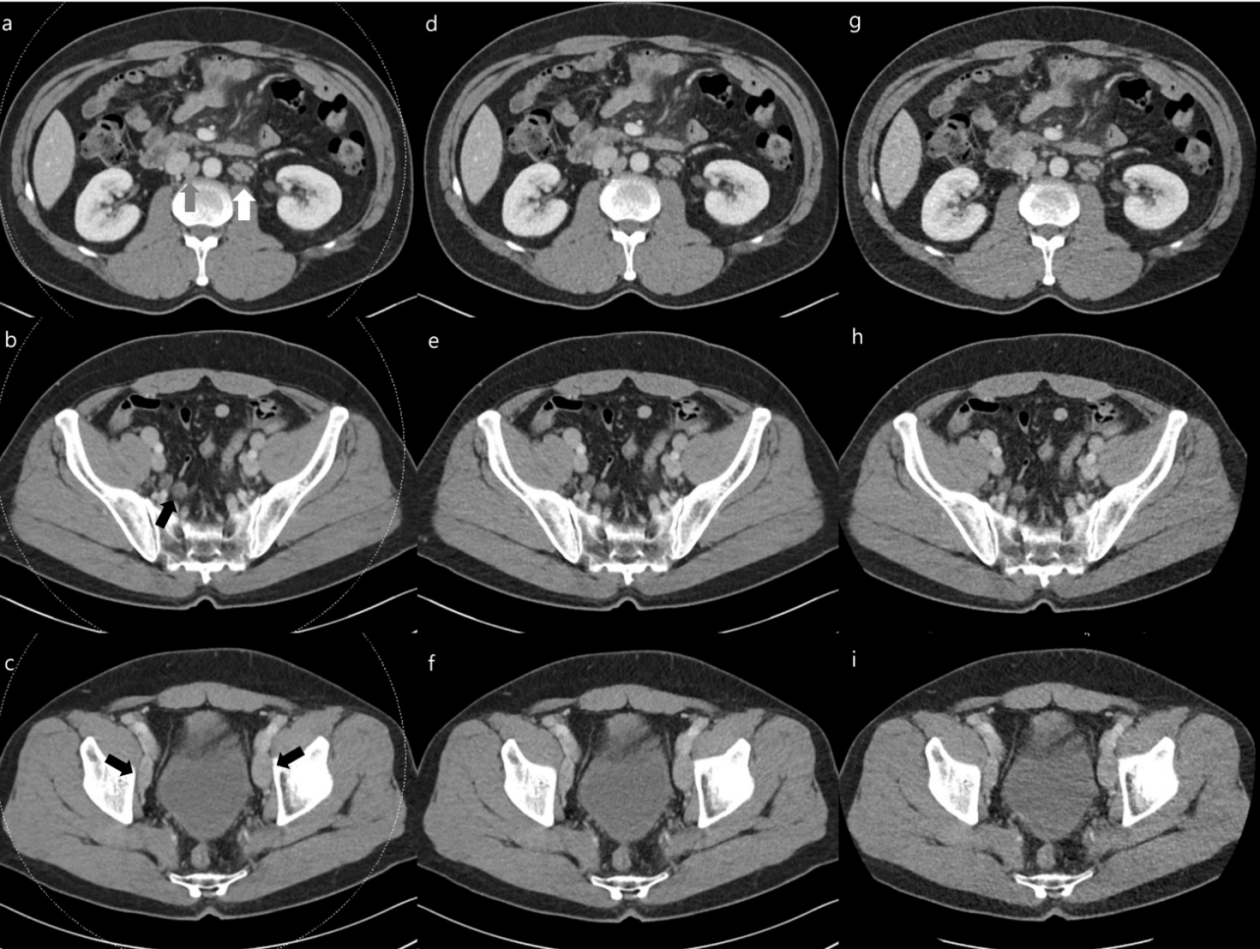
Abdominopelvic computed tomography (CT) images of a 54-year-old man with follicular lymphoma, partial response state (body mass index, 29.4 kg/m^2^; effective diameter, 27.0 cm). The three different types of CT images, acquired according to the radiation dose (a-c: standard-dose CT, 6.8 mSv; d-f: low-dose CT, 4.5 mSv; g-i: ultralow-dose CT, 2.3 mSv), show multiple enlarged paraaortic (white arrow), aortocaval (gray arrow), right common iliac, and bilateral external iliac lymph nodes (black arrows). Identical findings for the aforementioned lesions were reported by two radiologists. The overall image quality score was 4 for ultralow-dose CT images and 5 for low-dose and standard-dose CT images, according to two radiologists; the image quality score was 5 for all the three types of CT images according to one hematologist.

Table 4 compares standard-dose, low-dose, and ultralow-dose CT for the detection of lesions in the aforementioned organs and enlarged lymph node analysis in patients with lymphoma. The number of enlarged lymph nodes detected by two readers was higher on standard-dose (n = 97 and 95) and low-dose CT (n = 97 and 95) than on ultralow-dose CT (n = 94 and n = 93). The enlarged lymph node-based detection rates (i.e., number of detected true enlarged lymph nodes/number of true enlarged lymph nodes) for two readers were 97.0% (96/99) and 94.0% (93/99) on standard-dose CT, 97.0% (96/99) and 94.0% (93/99) on low-dose CT, and 94.0% (93/99) and 89.9% (89/99) on ultralow-dose CT (Table 4A and Figs 2 and 3). After GEE with adjustment for multiple comparisons using Bonferroni correction, there was no statistical difference among three data sets by two readers (reader 1, Standard-dose CT vs. Ultralow-dose CT, *P* = 0.256; Low-dose CT vs. Ultralow-dose CT, *P* = 0.256; reader 2, Standard-dose CT vs. Ultralow-dose CT, *P* = 0.132; Low-dose CT vs. Ultralow-dose CT, *P* = 0.132). Pairwise comparisons revealed different numbers of enlarged lymph nodes in the common hepatic, paraaortic, aortocaval, and internal iliac areas (standard-dose CT vs. ultralow-dose CT and low-dose CT vs. ultralow-dose CT) among the three CT doses (Tables 4B and S1). The highest number of nodules in the adrenal glands was observed on standard-dose CT (n = 5, both), followed by low-dose CT (n = 3 and 4) and ultralow-dose CT (n = 3 and 2) by two readers.

**Table 4:** Lesion detection in organs (a) and detailed enlarged lymph node (b) analysis.

**(a) Number of detected lesions in organs**							
		Standard-dose CT	Low-dose CT		Ultralow-dose CT		Reference standard
• Enlarged lymph node*							99
Lymph node based							
**Reader 1**							
Detection		97	97		94		
False positive		1	1		1		
False negative		3	3		6		
Detection rate (%)		97.0 (96/99)	97.0 (96/99)		94.0 (93/99)		
**Reader 2**							
Detection		95	95		91		
False positive		2	2		2		
False negative		6	6		10		
Detection rate (%)		94.0 (93/99)	94.0 (93/99)		89.9 (89/99)		
Examination based							26
Reader 1		26	26		26		
Reader 2		26	26		26		
• Liver lymphoma involvement							0
Reader 1		0	0		0		
Reader 2		0	0		0		
• Nodule in adrenal glands							5
Reader 1		5	3		3		
Reader 2		5	4		2		
• Splenomegaly							
Reader 1		20	22		23		
Reader 2		24	24		22		
**(b) Number of enlarged lymph nodes**							
	Standard-dose CT			Low-dose CT		Ultralow-dose CT	
Left gastric area							
Reader 1	5			5		5	
Reader 2	5			5		5	
Common hepatic area							
Reader 1	8			8		7	
Reader 2	9			9		8	
Portocaval area							
Reader 1	6			6		6	
Reader 2	7			7		7	
Retrocrural area							
Reader 1	2			2		2	
Reader 2	2			2		2	
Paraaortic area							
Reader 1	17			17		17	
Reader 2	15			15		14	
Aortocaval area							
Reader 1	14			14		13	
Reader 2	11			11		10	
Common iliac area							
Reader 1	15			15		15	
Reader 2	16			16		16	
Internal iliac area							
Reader 1	4			4		3	
Reader 2	5			5		4	
External iliac area							
Reader 1	26			26		26	
Reader 2	25			25		25	

The number of patients with splenomegaly on ultralow-dose, low-dose, and standard-lose CT was determined to be 23, 22, and 23 for radiologist 1; 22, 24, and 24 for radiologist 2; and 16, 16, and 18 for the hematologist, respectively. Spleen size measured by three readers (radiologists and one hematologist was 10.7 ± 1.69, 10.6 ± 1.78, and 10.0 ± 0.28 cm on ultralow-dose CT; 10.7 ± 1.70, 10.7 ± 1.80, and 10.0 ± 0.14 on low-dose CT; and 10.7 ± 1.70, 10.7 ± 1.79, and 10.0 ± 0.28 cm on standard-dose CT, respectively). We measured the average diameter of lymph nodes (short-axis diameter >0.5 cm, a total of 170 lymph nodes in each data set) on standard-dose, low-dose, and ultralow-dose CT, with no significant differences in the mean lymph node diameter among them (0.7±0.3, 0.7±0.3, and 0.7±0.4, respectively).

## Quantitative and qualitative image analysis

A comparison of objective CT image quality based on measurement of image noise in subcutaneous fat, psoas muscle, liver, and abdominal aorta is presented in Table 5. Standard-dose CT exhibited the lowest image noise (9.4–11.5 HU), followed by low-dose CT (11.0–14.3 HU) and ultralow-dose CT (12.2–15.8 HU; *P*-value < 0.001; all post-hoc analysis). With regard to subjective image quality assessed by all readers, ultralow-dose CT exhibited lower overall image quality (3.8–4.1; *P* < 0.001; Kruskal-Wallis test), while standard-dose CT (4.7–4.9) and low-dose CT (4.6–4.8) exhibited higher overall image quality. There was no significant difference in overall image quality between standard-dose and low-dose CT (*P*-value [adjusted with Monte Carlo simulation] = 0.167, 0.121, and 0.088 for each reader, respectively).

**Table 5:** Quantitative and qualitative image analysis of three CT image sets.

	Standard-dose CT	Low-dose CT	Ultralow-dose CT	*P*-value
Quantitative analysis (Hounsfield unit, HU)				
Noise				
Subcutaneous fat	9.4 ± 3.1	11.0 ± 3.1	12.2 ± 3.6	< 0.001
Psoas muscle	10.5 ±1.8	13.2 ± 2.6	14.5 ± 2.9	< 0.001
Liver	10.3 ± 2.0	12.7 ± 2.8	14.7 ± 2.7	< 0.001
Abdominal aorta	11.5 ± 2.9	14.3 ± 3.0	15.8 ± 3.3	< 0.001
Attenuation				
Subcutaneous fat	-113.8 ± 9.0	-113.8 ± 9.1	-112.1 ± 9.4	< 0.001
Psoas muscle	63.1 ± 7.6	63.5 ± 7.2	63.2 ± 7.2	< 0.001
Liver	122.0 ± 18.7	123.2 ± 18.9	120.6 ± 19.4	< 0.001
Abdominal aorta	188.9 ± 31.1	189.3 ± 31.3	190.4 ± 38.9	< 0.001
Overall image quality*				
Reader 1	4.8 ± 0.4	4.7 ± 0.4	3.8 ± 0.5	< 0.001
Reader 2	4.7 ± 0.4	4.6 ± 0.5	3.9 ± 0.5	< 0.001
Reader 3	4.9 ± 0.2	4.8 ± 0.3	4.1 ± 0.5	< 0.001

*Readers 1 and 2 were both radiologists, and reader 3 was a hematologist.

### Inter-observer agreement

S1 Table presents the inter-reader agreement of enlarged lymph node detection. Inter-reader agreement for enlarged lymph node detection by the two readers was excellent (κ *=* 0.83–1). Enlarged aortocaval lymph nodes on ultralow-dose CT exhibited the lowest inter-reader agreement score (κ *=* 0.83) in both common iliac areas. Intraclass correlation coefficient of spleen size was 0.90 (95% confidence interval [CI]), 0.87–0.93), 0.91 (95% CI, 0.88–0.94), and 0.91 (95% CI, 0.88–0.93) on ultralow-dose, low-dose, and standard-dose CT, respectively.

### Radiation dose parameters

Table 6 summarizes the dose parameters of the three CT image sets. Mean CTDI_vol_ of standard-dose, low-dose, and ultralow-dose CT was 5.6 ± 1.5 mGy, 3.7 ± 1.0 mGy, and 1.9 ± 0.5 mGy, respectively. Mean effective radiation dose of standard-dose, low-dose, and ultralow-dose CT was 5.7 ± 1.8 mSv, 3.8 ± 1.2 mSv, and 1.9 ± 0.6 mSv, respectively.

**Table 6:** Dose parameters of three CT image sets.

	Standard-dose CT	Low-dose CT	Ultralow-dose CT	*P*-value
CTDI_vol_ (mGy)	5.6 ± 1.5 (2.5–11.0)	3.7 ± 1.0 (1.7–7.4)	1.9 ± 0.5 (0.8–3.6)	< 0.001
Dose-length product (mGy-cm)	335.7 ± 108.4 (137.9–710.1)	223.9 ± 72.3 (92.0–473.6)	72.5 ± 20.1 (45.9–236.5)	< 0.001
Effective dose (mSv)	5.7 ± 1.8 (2.3–12.1)	3.8 ± 1.2 (1.5–8.1)	1.9 ± 0.6 (0.8–4.0)	< 0.001

## Discussion

This study compared the radiation dose exposure, the detection rate of enlarged lymph nodes, and image quality of standard-dose, low-dose, and ultralow-dose abdominopelvic CT using the ADMIRE algorithm in patients with lymphoma. The enlarged lymph node-based detection rate was 94.0−97.0% on standard-dose and low-dose CT, and 89.9−94.0% on ultralow-dose CT according to two readers, with excellent inter-reader agreement. Ultralow-dose and low-dose CT effectively reduced radiation dose by 66.7% and 33.3%, respectively, while maintaining adequate image quality. We observed the same rate of detection of enlarged lymph nodes (i.e., > 1 cm in diameter) by two readers between low-dose and standard-dose CT. Although the rate of detection of enlarged lymph nodes was slightly higher on standard-dose CT than on ultralow-dose CT, there was no statistically significant difference between standard-dose and ultralow-dose CT. We conjecture that the detection of enlarged abdominal lymph nodes can be achieved in a relatively simple and clear manner by radiologists. The development of CT techniques and image reconstruction algorithms, including ADMIRE, may facilitate higher detection rates, even on ultralow-dose CT.

Several studies have reported non-inferior or comparable diagnostic performance for a specific diagnosis (e.g., urinary stones or acute appendicitis) of a relatively simple disease or organ between low-dose abdominal CT and standard-dose CT [23–25]. However, the diagnostic performance of low-dose CT for small or inconspicuous abdominal structures has been unsatisfactory, even with the use of model-based IR [26, 27]. Due to high image noise in the abdomen, low-dose CT has limitations in the evaluation of small lesions in neoplastic conditions (e.g., liver metastasis and pancreatic cancer) and inflammatory diseases [18, 27, 28]. Small lesions with high image noise may obscure lesion detection due to similar attenuation as the background [27, 29]. Compared to standard-dose CT, ultralow-dose CT exhibited a similar rate of detection of enlarged lymph nodes in this study, while reducing the radiation dose by 33.3%. The high rate of detection of enlarged lymph nodes on ultralow-dose CT could be underpinned by the abdominal lymph nodes being less affected by image noise due to definite contrast differences between retroperitoneal or peritoneal fat and surrounding lymph nodes with a clear margin (i.e., background and lesion sharpness). These findings suggest that ultralow-dose CT may be employed instead of standard-dose CT during follow-up of patients with lymphoma. Notably, the interobserver agreement for abdominal lymph nodes was excellent in our study. This could be due to the sparsity of other anatomical structures surrounding the abdominal lymph nodes; as such, the diagnosis of enlarged lymph nodes exhibited small differences between the two readers despite the presence of image noise.

With regard to lymph node evaluation using low-dose CT, Paolini et al. reported that there was no significant difference in the delineation of thoracic lymph nodes between contrast-enhanced low-dose and standard-dose CT [30]. Mueller‑Lisse et al. also reported that contrast-enhanced low-dose CT with approximately 1 mSv revealed equivalent delineation of thoracic lymph nodes compared to standard-dose CT [31]. In line with prior studies on thoracic lymph nodes, our study revealed similar results regarding abdominal lymph node evaluation using low-dose and ultralow-dose CT in patients with lymphoma.

Hérin et al. reported that reduced-dose CT with model-based IR could reduce the amount of radiation delivered to patients with lymphoma while maintaining image quality comparable to that of standard-dose CT with filtered-back projection [12]. Herein, we compared three CT image sets using ADMIRE (i.e., without using a filtered-back projection comparison). We implemented ADMIRE in actual practice and focused on the number of radiation doses to be reduced in patients with lymphoma while maintaining image quality. Quantitative image noise increased with a decrease in radiation dose. However, we observed that subjective visual image quality was comparable between standard-dose and low-dose CT, and there were no significant differences in the results of lymph node evaluation between these image sets. Although low-dose CT exhibited slightly increased objective image noise and comparable subjective image quality compared to standard-dose CT, this may not affect the diagnosis of lymph node detection. Nevertheless, ultralow-dose CT resulted in degradation of both qualitative visual image quality and quantitative image noise analysis.

A blind image analysis was performed in this study for the independent evaluation of lymph node enlargement in each image reading session. The use of low-dose CT as an imaging modality for follow-up evaluation of lymphoma permits the use of initial standard-dose CT as a reference and comparative examinations in actual practice. We predict better diagnostic performance of enlarged lymph node detection, even using ultralow-dose CT, compared to the current results. In consideration of the long-term follow-up period, low-dose and/or ultralow-dose CT offers a safe and accurate alternative imaging modality to replace current standard-dose CT.

Our study has a few limitations. First, we only assessed changes in tube load (mAs) and a single image reconstruction using ADMIRE, and we did not consider filtered-back projection. Thus, the study protocol differs from clinical protocols, as a fixed kVp was used for both detection tubes without automatic tube voltage adjustment (Care kV). As various image acquisition parameters can affect image quality in CT, our results may have limited generalizability. Second, as images reconstructed with ADMIRE have different appearances, it is difficult to achieve a true blinded analysis of subjective criteria. Third, because most of the patients who underwent dual energy CT examination at our hospital were outpatients, most of the patients with lymphoma included in the study were outpatients, and we predominantly focused on treatment response. Therefore, our results may be applicable to surveillance of patients after lymphoma treatment but not for patients with initial lymphoma evaluation, relapse, or suspected progressive disease. Finally, we analyzed CT images with a slice thickness of 5 mm in the axial direction and 3 mm in the coronal direction. Although these parameters can reflect readings in actual practice, it may also lead to partial volume effect when measuring lymph nodes since thin slices (e.g., 2 mm) were not analyzed.

In conclusion, our findings suggest that ultralow-dose and low-dose CT effectively reduce radiation dose by 66.7% and 33.3%, respectively, while maintaining acceptable image quality and can be used as an imaging modality for follow-up evaluation of lymphoma, in consideration of the long-term follow-up period.

## Supporting information

S1 Table The number of enlarged lymph nodes and each interobserver agreement. (DOCX)

S1 Appendix STROBE checklist. (DOC)

S2 Appendix Statistical analysis of subjective image quality comparisons. (DOCX)
