# Supplementary material for: Low-dose abdominopelvic computed tomography in patients with lymphoma: An image quality and radiation dose reduction study
Source: PLoS One. 2022 Aug 11;17(8):e0272356. doi: 10.1371/journal.pone.0272356 (PMC9371255; doi:10.1371/journal.pone.0272356)
Supplement: S1 Table — (DOCX) [file pone.0272356.s001.docx]

Supplementary table 1. The number of enlarged lymph nodes and each interobserver agreement

|  | Standard-dose CT | Low-dose CT | Ultralow-dose CT |  | κ |  |
| --- | --- | --- | --- | --- | --- | --- |
|  |  |  |  | Standard-dose | Low-dose | Ultralow-dose |
| Left gastric area |  |  |  | 1 | 1 | 1 |
| Reader 1 | 5 | 5 | 5 |  |  |  |
| Reader 2 | 5 | 5 | 5 |  |  |  |
| Common hepatic area |  |  |  | 1 | 1 | 1 |
| Reader 1 | 8 | 8 | 7 |  |  |  |
| Reader 2 | 9 | 9 | 8 |  |  |  |
| Portocaval area |  |  |  | 0.92 | 0.92 | 0.92 |
| Reader 1 | 6 | 6 | 6 |  |  |  |
| Reader 2 | 7 | 7 | 7 |  |  |  |
| Retrocrural area |  |  |  | 1 | 1 | 1 |
| Reader 1 | 2 | 2 | 2 |  |  |  |
| Reader 2 | 2 | 2 | 2 |  |  |  |
| Paraaortic area |  |  |  | 0.93 | 0.93 | 0.89 |
| Reader 1 | 17 | 17 | 17 |  |  |  |
| Reader 2 | 15 | 15 | 14 |  |  |  |
| Aortocaval area |  |  |  | 0.87 | 0.87 | 0.86 |
| Reader 1 | 14 | 14 | 13 |  |  |  |
| Reader 2 | 11 | 11 | 10 |  |  |  |
| Common iliac area |  |  |  | 0.87 | 0.87 | 0.83 |
| Reader 1 | 15 | 15 | 15 |  |  |  |
| Reader 2 | 16 | 16 | 16 |  |  |  |
| Internal iliac area |  |  |  | 1 | 1 | 0.86 |
| Reader 1 | 4 | 4 | 3 |  |  |  |
| Reader 2 | 5 | 5 | 4 |  |  |  |
| External iliac area |  |  |  | 0.94 | 0.94 | 0.94 |
| Reader 1 | 26 | 26 | 26 |  |  |  |
| Reader 2 | 25 | 25 | 25 |  |  |  |
