# Supplementary material for: Low-dose abdominopelvic computed tomography in patients with lymphoma: An image quality and radiation dose reduction study
Source: PLoS One. 2022 Aug 11;17(8):e0272356. doi: 10.1371/journal.pone.0272356 (PMC9371255; doi:10.1371/journal.pone.0272356)
Supplement: S2 Appendix — (DOCX) [file pone.0272356.s003.docx]

|  |  |  |  |  |  |  |
| --- | --- | --- | --- | --- | --- | --- |
| **Source** | **DF** | **Anova SS** | **Mean Square** | **F Value** | **Pr > F** |  |
| **test_no** | 2 | 77.811024 | 38.90551181 | 219.01 | <.0001 | between 3tests |
| **test_no** | 1 | 0.3188976 | 0.31889764 | 1.93 | 0.1659 | between 2tests (standard vs low) |
|  |  |  |  |  |  |  |
|  |  |  |  |  |  |  |
|  |  |  |  |  |  |  |
| **Type 3 Tests of Fixed Effects** | | | | |  |  |
| **Effect** | **Num DF** | **Den DF** | **F Value** | **Pr > F** |  |  |
| **test_no** | 2 | 377 | 280.32 | <.0001 | between 3tests |  |
| **test_no** | 1 | 251 | 2.42 | 0.1208 | between 2tests (standard vs low) |  |
|  |  |  |  |  |  |  |
|  |  |  |  |  |  |  |
| **Source** | **DF** | **Anova SS** | **Mean Square** | **F Value** | **Pr > F** |  |
| **test_no** | 2 | 60.204724 | 30.1023622 | 123.09 | <.0001 | between 3tests |
| **test_no** | 1 | 0.6653543 | 0.66535433 | 2.94 | 0.0875 | between 2tests (standard vs low) |
|  |  |  |  |  |  |  |
|  |  |  |  |  |  |  |
| **Type 3 Tests of Fixed Effects** | | | | |  |  |
| **Effect** | **Num DF** | **Den DF** | **F Value** | **Pr > F** |  |  |
| **test_no** | 2 | 377 | 143.88 | <.0001 | between 3tests |  |
| **test_no** | 1 | 251 | 3.45 | 0.0645 | between 2tests (standard vs low) |  |
|  |  |  |  |  |  |  |
|  |  |  |  |  |  |  |
|  |  |  |  |  |  |  |
| **Source** | **DF** | **Anova SS** | **Mean Square** | **F Value** | **Pr > F** | between 3tests |
| **test_no** | 2 | 46.367454 | 23.18372703 | 166.51 | <.0001 | between 2tests (standard vs low) |
| **test_no** | 1 | 0.476378 | 0.47637795 | 5.87 | 0.0161 |  |
|  |  |  |  |  |  |  |
|  |  |  |  |  |  |  |
| **Type 3 Tests of Fixed Effects** | | | | |  |  |
| **Effect** | **Num DF** | **Den DF** | **F Value** | **Pr > F** |  |  |
| **test_no** | 2 | 377 | 170.21 | <.0001 | between 3tests |  |
| **test_no** | 1 | 251 | 5.94 | 0.0155 | between 2tests (standard vs low) |  |
